# Blackberry Fruit Classification in Underexposed Images Combining Deep Learning and Image Fusion Methods

**DOI:** 10.3390/s23239543

**Published:** 2023-11-30

**Authors:** Eduardo Morales-Vargas, Rita Q. Fuentes-Aguilar, Emanuel de-la-Cruz-Espinosa, Gustavo Hernández-Melgarejo

**Affiliations:** 1 Tecnologico de Monterrey, Institute of Advanced Materials for Sustainable Manufacturing, Av. Gral Ramón Corona No 2514, Colonia Nuevo México, Zapopan 45201, Jalisco, Mexico; emoralesv@tec.mx (E.M.-V.); g_hernandezm@tec.mx (G.H.-M.); 2 Tecnologico de Monterrey, Escuela de Ingeniería y Ciencias, Av. Gral Ramón Corona No 2514, Colonia Nuevo México, Zapopan 45201, Jalisco, Mexico; a01150821@tec.mx

**Keywords:** blackberry classification, ripeness stage classification, feature fusion, classification methods

## Abstract

Berry production is increasing worldwide each year; however, high production leads to labor shortages and an increase in wasted fruit during harvest seasons. This problem opened new research opportunities in computer vision as one main challenge to address is the uncontrolled light conditions in greenhouses and open fields. The high light variations between zones can lead to underexposure of the regions of interest, making it difficult to classify between vegetation, ripe, and unripe blackberries due to their black color. Therefore, the aim of this work is to automate the process of classifying the ripeness stages of blackberries in normal and low-light conditions by exploring the use of image fusion methods to improve the quality of the input image before the inference process. The proposed algorithm adds information from three sources: visible, an improved version of the visible, and a sensor that captures images in the near-infrared spectra, obtaining a mean F1 score of 0.909±0.074 and 0.962±0.028 in underexposed images, without and with model fine-tuning, respectively, which in some cases is an increase of up to 12% in the classification rates. Furthermore, the analysis of the fusion metrics showed that the method could be used in outdoor images to enhance their quality; the weighted fusion helps to improve only underexposed vegetation, improving the contrast of objects in the image without significant changes in saturation and colorfulness.

## 1. Introduction

The agriculture industry grows annually in several ways to meet human needs. For example, according to the Mexican government agency Secretaría de Agricultura, Ganadería, Desarrollo Rural, Pesca y Alimentación (SAGARPA), berry crops (blackberry, raspberry, blueberry, cranberry, strawberry) along the Mexican territory reported annual increases close to 22% between 2003 and 2016 [1], reaching up to 390,239 tons produced at the end of 2017. The harvesting tasks required to collect large production volumes are mainly based on manual labor, requiring an increase proportional to industry growth for sustainable production [2]. However, unlike this requirement, the labor force arriving in this industrial sector exhibits a decreasing trend, causing significant losses of up to 12% of the berries produced, mainly because the shelf life of the harvested berries is shorter than that of other types of fruits [3,4]. This is not an isolated problem or exclusive to the production of berries in Mexico, but a continuous trend around the world that was exacerbated by the COVID-19 travel restrictions [5,6,7,8]. Monetary losses due to spoiled fruit that is not harvested are a solid motivation to implement innovative processes in the agriculture industry. For example, among the enabling technologies of precision and smart agriculture, robotic systems have been considered as one of the most promising approaches to address labor shortages for seeding, fertilizing, and harvesting tasks [9,10,11]. Robotic systems for agriculture could be implemented with unmanned aerial vehicles, unmanned ground vehicles, manipulator arms, or as subsystems used in harvesting [12]. Such subsystems consider specific developments in the gripper, vehicle, control strategies, navigation systems, and artificial vision systems.

One of the most significant issues within automated harvesting lies in artificial vision systems, as the predominant problem is the variable illumination conditions that affect the detection and identification of fruits and the maturity stages. Illumination problems affect the harvest success rate because the robot must be able to locate fruit in challenging environments [13,14]. Furthermore, maturity identification is severely affected, as berries do not ripen during storage and transportation periods after picking, which can cause the delivery of unripe fruit [15]. In fact, the detection and classification of fruits in underexposed images and low-light conditions is a challenge that is not commonly addressed in the literature; most work assumes normal conditions in greenhouses and orchards without controlled light [16,17,18].

However, the enhancement of low-light conditions, underexposed images, and visibility problems such as fog are more commonly addressed in image processing, independently of the application domain, in which common approaches are image fusion and image enhancement [19,20,21]. Furthermore, the use of algorithms that enhance images independently if they fuse information from different sources finds applications in a wide range of fields, including fruit ripeness classification, surveillance, aerial photography for vegetation analysis, autonomous navigation, and biomedical applications, among others [22,23,24]. For example, focused on low-illumination changes, Dong et al. proposed an algorithm to improve low illumination in videos based on a modification of a dehazing algorithm [20] and Hau Ngo et al. use a nonlinear expansion function to fuse images’ Visible Spectrum (VIS) and Near Infra Red (NIR), obtaining a more intense image for dark zones while reducing the light intensity in overexposed regions [25]. On the other hand, the current work that fuses information from different sources aims to extract information such as texture, edges, or light quantity to improve the details of the VIS image by adding these characteristics or combining several filters such as edge-preserving, bilateral, and disparity filters. Bennett et al. employ a linear mapping of luminance and extracted features from IR spectra to reduce image noise, improve image sharpness, and improve edge definition in the image while preserving the original illumination level within the VIS image [26]; however, the algorithm produces color changes after processing, which leads to another challenge: color reconstruction. Vanmali et al. 2015 use a light transmission model to recover light scattered from the NIR [19]. On the other hand, Vanmali et al. 2017 use a Laplacian–Gaussian pyramid filter and multiresolution fusion to improve image visibility [27]. The main drawback in these works is the oversaturation of colors in the image, generating fused images with unreal colors. However, Herrera addressed the problem in his works Herrera et al. 2019 and Herrera et al. 2021 by fusing the information into the luminance space and adding only the information extracted from morphological operations, such as the top-hat transform [21,28]. Furthermore, although some works fuse the benefits of both approaches (Mohamed et al. 2019), long processing times are a limitation in real-time applications [29].

Therefore, in this work, a fusion method is proposed that focuses on adding vegetation-related information to the image to complement visual information with those in the NIR band to improve visualization by improving the contrast between the background and blackberries. The process uses Normalized Difference Vegetation Index (NDVI) to enhance objects in the scene, adaptively focusing on fruits and vegetation. As a result, the method produces an enhanced image with more contrast between regions of low-contrast objects, resulting in a more effective classification between ripe and unripe blackberries in uncontrolled light conditions. The results obtained with the proposed method include a reduction in processing times compared to the methods in the literature and improvements in contrast and colorfulness features while maintaining saturation in low-exposure images. The remainder of the paper is as follows. Section 2 presents the materials and methods considered for this work, including the theoretical basis required for the evaluation of fusion methods and image classification models, as well as the proposal to deal with blackberry classification in uncontrolled light environments. Later, in Section 3, the experimental setup is explained in detail and the corresponding results are presented and analyzed. Finally, Section 4 presents the conclusions and future work.

## 2. Materials and Methods

This section presents the theoretical framework required to understand the development of the proposed algorithm; it is divided into two major parts. The first part introduces the evaluation metrics of image fusion and the last section explains the proposed fusion method, tested on images acquired in blackberry greenhouses with low- and normal-light conditions.

### 2.1. Evaluation Metrics of Image Fusion Methods

Image fusion consists of merging two or more images acquired with different sensors, i.e., one in the VIS and the other in the NIR spectrum. The main objective is to improve the visualization of objects in a scene with low visibility (contrast) by adding new information and characteristics from one image to another [29,30]. For example, when information from the NIR spectrum is added (σ=[700−800] nm) to a VIS image, the visibility of objects in the background affected by fog can be improved [19,21]. The evaluation of image improvement using image fusion methods in the literature is usually performed using four metrics: Contrast (*C*), Entropy (EN), Colorfulness (CF), and Saturation (*S*). Here, we add the definition of each evaluation metric to improve the reproducibility of the results and avoid ambiguities because, for example, C can be defined in different ways according to the context or the author. C measures the difference between regions in the image, translated into better discrimination between the background and regions of interest (ROIs) by Equation (1), where I(i,j) is the intensity of the pixel image, and *n* and *m* are the width and height of the image, respectively [31]

(1) $$C=\frac{1}{nm}\sum_{i=1}^{n}\sum_{j=1}^{m}I{\left(i,j\right)}^{2}-{\left(\frac{1}{nm}\sum_{i=1}^{n}\sum_{j=1}^{m}I\left(i,j\right)\right)}^{2}.$$

Similarly to *C*, EN measures the information added to the fused image from NIR but from another perspective. In this case, a low value of EN means less information, and higher values of EN mean more information is added from the NIR to the VIS images. The EN is calculated using the cumulative frequency of the gray values over each color channel by Equation (2), where P(g) is the probability of a pixel with gray level *g*, and *G* is the maximum possible gray level, 255 in the case of an 8-bit image [32,33]

(2) $$EN=-\sum_{g=0}^{G}P\left(g\right)\log_{2}\left(P\left(g\right)\right).$$

On the other hand, the CF helps to measure the color variation by quantifying the chrominance information after image fusion [33,34]. In this manner, assigning an attribute that a human being can recognize is possible. Therefore, higher values of CF are expected compared to the original image. In addition, a qualitative analysis confirms that the fusion method does not change or oversaturate the original colors. However, color distortion is a usual effect after fusion due to the addition of new information [35]. This evaluation metric starts by converting the RGB image to the CIE L*a*b* color space, and then Equation (3) calculates the colorfulness of the image by using the red/green (a*) and the yellow/blue (b*) coordinates [36,37]. The terms $\sigma_{a}$ and $\sigma_{b}$ are the standard deviations along the color axes a* and b*, respectively, and *m* and *n* are the number of rows and columns of the fused image *I*.

(3) $$CF=\sqrt{\sigma_{a}+\sigma_{b}}+0.94\left(\frac{1}{mn}\sum_{i=1}^{m}\sum_{j=1}^{n}\sqrt{a{\left(i,j\right)}^{2}+b{\left(i,j\right)}^{2}}\right).$$

Finally, *S* in an image measures the intensity or depth of color; a very brightly colored image has a high saturation value. On the contrary, low values of *S* mean that the image appears to be shaded, without brightness [31]. However, the value must be maintained as close as possible to the original image value to avoid color over-saturation or under-saturation. The average *S* is obtained using Equation (4), where S(i,j) is the saturation channel of the Hue Saturation Intensity (HSI) color space, and *m* and *n* are the number of rows and columns of the fused image *I*.

(4) $$S=\frac{1}{mn}\sum_{i=1}n\sum_{j=1}^{n}S\left(i,j\right).$$

### 2.2. VIS-NIR Image Fusion

**Figure 1:**
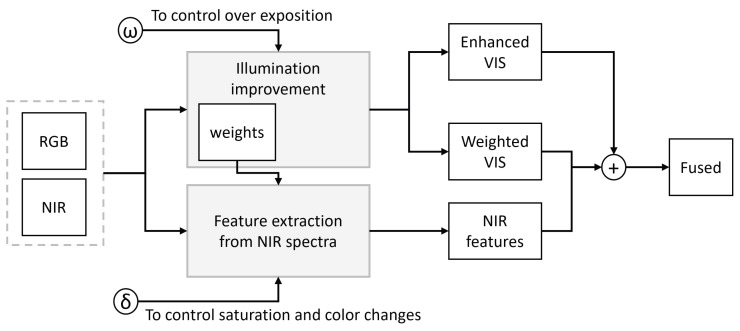
Flowchart of the methodology to fuse information from the NIR and VIS spectra while the illumination is improved.

Most classifiers of ripeness states do not consider low-light or poor-exposure conditions. This hole in the literature motivated us to improve the current classification rates of models that may have problems with fruits like blackberries because their dark color can be confused with the background. Therefore, this work focuses on improving blackberry classification rates under low-light and normal-light conditions. It was hypothesized that a fusion method that includes information from the VIS and NIR spectral bands and an image enhancement process can improve the quality of underexposed images and thus improve classification rates, providing evidence of how a vision system of a robotic arm can be more robust in such circumstances. The proposal comprises two modules. The first is an illumination improvement and establishes weights that can control the information portions that can be transferred from NIR (Figure 1). The modified algorithm uses NDVI to control the improvement of light, in addition to overall illumination, to improve the exposition of vegetation, while the illumination in the upper sections of the greenhouse does not become overexposed [38]. The second stage uses the dark channel from the first stage, the VIS and the NIR image, to add information that the VIS sensor does not capture. This includes shapes and other information related to biological processes such as photosynthesis [39,40,41].

**Figure 2:**
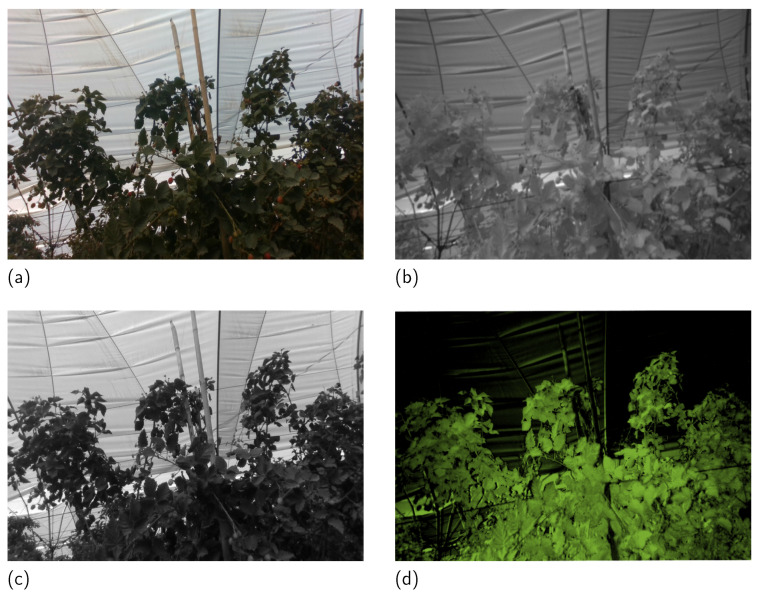
Example of an NDVI map calculated from an image taken in the greenhouse. (**a**) VIS image, (**b**) NIR image, (**c**) red channel of the input image, and (**d**) VI with a false color from dark to green in the range [−1,1].

The method starts by calculating the Vegetation Index (VI) or NDVI defined in Equation (5), where *N* is the light reflected in the NIR band and *R* the light in the red channel of a camera in the VIS spectrum. The NDVI is an index commonly used in the agriculture industry and remote sensing to correlate reflected light in the red and NIR spectra to measure the photosynthetic activity of plants. In this work, it is applied to NDVI to adaptively improve the image based on vegetation (ROI). In mathematical terms, NDVI is the relation between the difference in the red and the NIR channels *R* and *N*, respectively, which are two grayscale images with values between 0 and 255. The results are in the range of [−1,1] where negative values are related to objects such as water, snow, or clouds, values near 0 to rocks, and values greater than 0.2 to vegetation. Figure 2 shows an NDVI map for reference.

(5) $$VI=\frac{N-R}{N+R}.$$

The next step consists of inverting *I* to obtain an image in which the inverted regions with low light are converted into saturated regions according to the dark channel prior algorithm to improve images with low quality [38]. Thus, the process consists of subtracting from each pixel of *I* the maximum possible values that the processed image can have ($2^{n}-1$). For example, for an image of 8 bits, the subtracting value must be $2^{n}=256-1$, minus 1, because 0 is counted as a possible value. The process is repeated for each channel *c*. Then, the complement of an image is defined mathematically in Equation (6), in which ${I\prime }^{c}$ is the complement image of the channel *c* of *I*, and *p* is a pixel of *I*.

(6) $${I\prime }_{p}^{c}=\left(2^{n}-1\right)-I_{p}^{c}.$$

**Figure 3:**
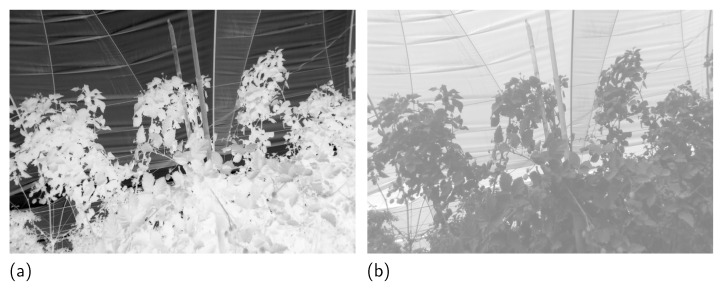
(**a**) Dark channel of the normalized light in the image, and (**b**) a transmission map that establishes how much the image will be improved.

Then the transmission map is calculated using the dark channel (D) of the normalized image. It is used to determine the intensity of the light improvement in the image and the quantity of the NIR information that will be fused with the VIS image. The first step involves the selection of the highest value of the VIS image from the 0.1% values with the lowest index of vegetation in the VI map. This process is repeated for each channel *c* and will represent the overall illumination of the scene (*L*). Then the dark channel *D* is calculated by obtaining the minimum intensity between channels of the normalized image as defined in Equation (8). Current works assume that the brightest pixels in the image must be the sky, but selecting the pixels with the lowest value from the VI map discards those that are vegetation. As seen in Figure 3, this process ensures the selection of pixels that belong to the sky or those pixels that do not belong to vegetation regions independently of the illumination in the scene. Then, the transmission map (*t*) is computed using the dark channel and the overall illumination using Equation (7), where ω is a parameter that controls the effect of improvement in the image, and D(p) is the minimum value between channels for a pixel *p*.

(7) $$t_{p}=\left(1-\omega \right)D_{p},$$

(8) $$D_{p}=\underset{c=\{r,g,b\}}{min}\frac{I_{p}^{c}}{L^{c}}.$$

The next step consists of enhancing the images by a certain amount considering the information from the transmission map by using Equation (9). The enhancing equation is based on a method to enhance low-lighting videos [38]. They propose a value *P* for each pixel *p* in the image that adjusts $t_{p}$ adaptively, assuming that high values of t(p) represent the background and values below 0.5 represent the ROIs. In this paper, the value of *P* is not considered, enhancing all the images equally without focusing on the sky or vegetation. However, the next step will alleviate this problem in a fusion process between the original, the enhanced, and the NIR image in order to reduce the oversaturation in well-exposed regions of the images, such as the sky, and improve the illumination in overexposed regions.

(9) $$R=L+\frac{I^{\prime }-L}{t}.$$

**Figure 4:**
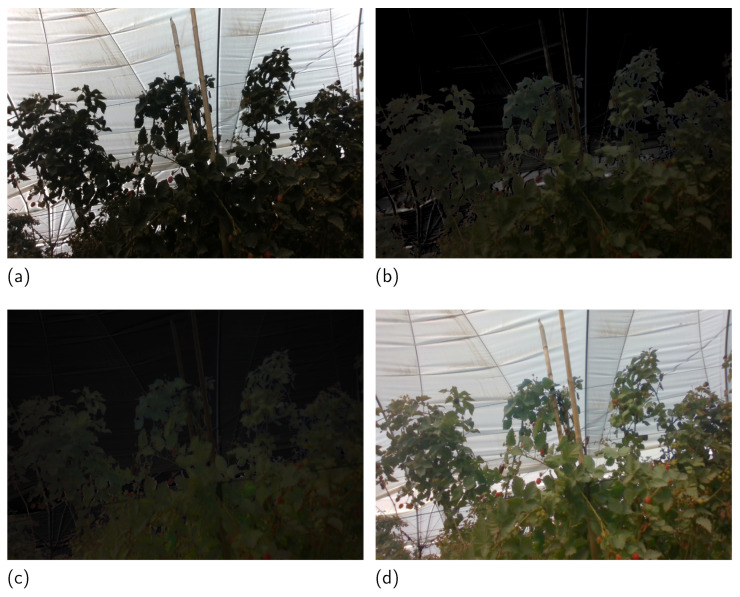
The output of the algorithm, with δ=0.7 and ω=0.5. The values were selected to improve the visualization without considering the quality of the output image. (**a**) RGB original image to fuse, (**b**) ERGB image, (**c**) FN image, and (**d**) fusion result of (**a**–**c**).

Finally, the information from the NIR spectra is fused to the VIS image. The image NIR is multiplied by an affectation δ that controls the amount of information it adds. The resulting image is multiplied by the relationship between the gray value of a particular color channel *c* and the sum of the values in the *r*, *g*, and *b* channels. This will maintain the color relationship in the added information, as seen in Figure 4c. Equation (12) defines the fusion step mathematically, and Figure 4d depicts the input, output, and added information, for reference. The complement of the vegetation index $VI^{\prime }$ is used to fuse the sky part of the original image, the enhanced vegetation region from the described steps, and the information from the NIR image. The fusion performed by Equation (13) generates images without overenhanced regions in the brightened part of the image and a better visualization of the berries in the vegetation.

(10) $$FN^{c}=\delta N*\left(D*\left[\frac{I^{c}}{I^{r}+I^{g}+I^{b}}\right]\right),$$

(11) $$FRGB_{p}^{c}=I*\left(1-\left[t_{p}^{c}*VI_{p}^{\prime }\right]\right),$$

(12) $$ERGB_{p}^{c}=R^{\prime }*\left(t_{p}^{c}*VI_{p}^{\prime }\right),$$

(13) $$F_{p}^{c}=FN_{p}^{c}+FRGB_{p}^{c}+ERGB_{p}^{c}.$$

## 3. Experiments and Results

The images for the validation of the proposed algorithm were acquired in a blackberry greenhouse in Ciudad Guzmán, the county seat of Zapotlán el Grande, located in the state of Jalisco, Mexico. Weather conditions such as temperature and sunlight were uncontrolled factors. However, to have different sunlight conditions, the images were taken between 11:00–13:00 and 17:00–19:00 to obtain images with normal- and low-light conditions and thus evaluate the performance of the proposed method under different illumination circumstances. Although the pictures were taken in a greenhouse facility, there was not artificial illumination and the sunlight intensity was not measured; the crops were not completely covered, since there was only a plastic ceiling. Therefore, some cameras and frameworks were tested to determine their performance and the feasibility of implementation according to the scope of the work. After an analysis, we selected the Intel^®^ RealSense^™^ Model D435f depth camera (Intel, Mountain View, CA, USA) for image acquisition. Its features include one RGB and two Infra Red (IR) sensors with an IR pass filter that improves depth quality performance by increasing the relative strength of the textured IR projector pattern. In addition, the filter helps alleviate visible reflections that transmit NIR light and absorb visible light, allowing it to work with solar light. A total of 370 registered image pairs with size 1920×1080 were taken, 185 with VIS and 185 with IR spectra. A tripod with a height of 135 cm was used to control height and angles (67.5${}^{\circ }$, 90${}^{\circ }$, and 112.5${}^{\circ }$) to visualize the fruits. Different distances from the camera and the plants were used, from 20 cm to 60 cm, with the same shot angles but horizontally. The images were processed on the MATLAB 2023a (The Mathworks, Inc., Natick, Massachusetts) platform due to the availability of state-of-the-art methods on a computer with a Ryzen 5 CPU at 3.6 Mhz and 32 GB RAM with an RTX 2060 Xtreme with 6 GB GDDR6 RAM and a core clock of 1845 MHz. Fusion methods were used with default parameters, and the proposed method used the values of δ=0.5 and ω=0.6, which were selected by qualitative analysis to improve visualization while maintaining image quality. The parameter δ improves the vegetation without significantly modifying the background. It improves the contrast between the leaves and the fruits. However, in values near 1, the edges blur. On the other hand, the ω parameter lightens the image, allowing visualization of dark areas without blurring, but also may generate an overexposure effect of the brightened areas.

**Figure 5:**
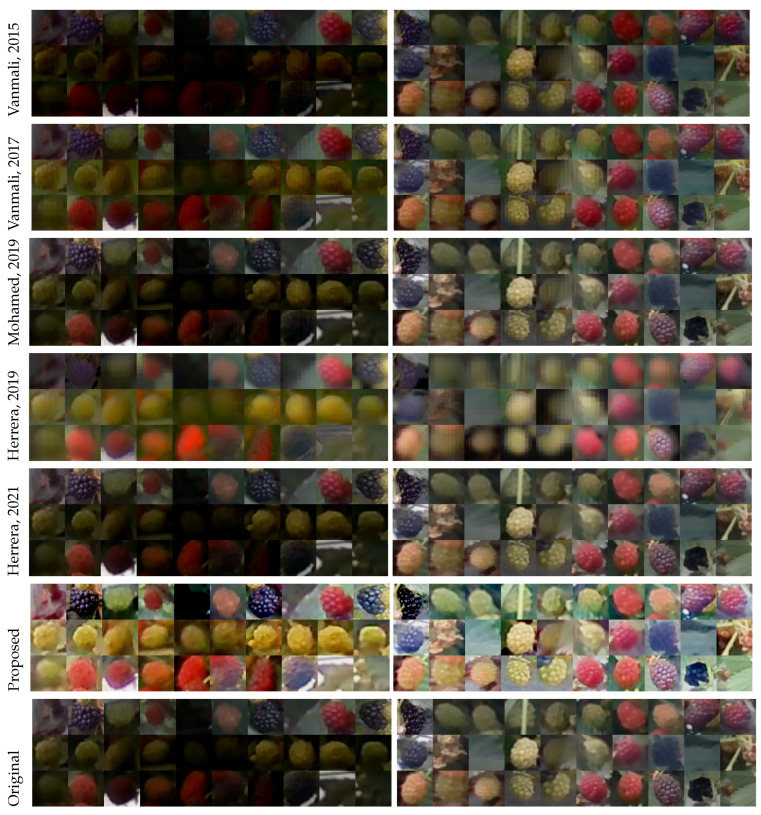
Comparison between fusion methods in state of the art and the proposed fusion and enhancement method. The methods that were compared are Vanmali et al. 2015 [19], Herrera et al. 2019 [21], Vanmali et al. 2017 [27], Herrera et al. 2021 [28], Mohamed et al. 2019 [29].

**Figure 6:**
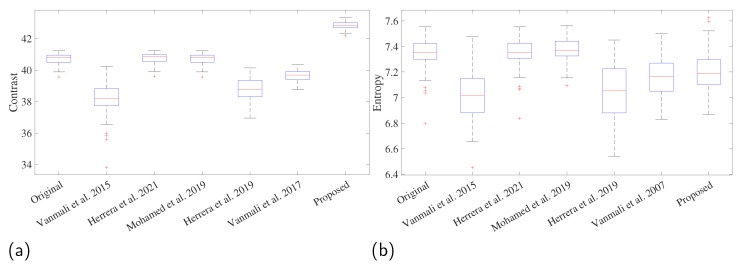
Comparison of image fusion quality metrics among methods. (**a**) Contrast and (**b**) entropy. The methods that were compared are Vanmali et al. 2015 [19], Herrera et al. 2019 [21], Vanmali et al. 2017 [27], Herrera et al. 2021 [28], Mohamed et al. 2019 [29].

The contrast, entropy, colorfulness, and saturation were calculated for all images in the dataset to compare the results between the proposed and reference methods. The comparison between the fusion and proposed methods is shown in representative images in Figure 5. The main objective of the fusion and enhancement method is to improve the differences between the berries and the background by combining information from three sources: the original image, an enhanced version, and information from the NIR band. The fusion of the three sources adapting the weight according to the atmospheric light means an improved contrast in the image in those images with low illumination; in other words, the difference between regions in the image may increase, which can be translated into a better visualization of the objects in the image for this kind of image. Thus, an increased contrast is expected while the entropy is maintained, since the proposed method increases the differences among regions without focusing on the details to maintain a low complexity. Comparisons were made using the Tukey multiple comparison test to compare the results with α=0.05. The measured contrast values shown in Figure 6 confirm the hypothesis that contrast increased significantly not only compared to the original image (difference = 2.083, *T*-value = −19.96, *p*-value = 0.00), but also compared to [19] (difference = 4.56, *T*-value = 0, *p*-value = 0) and [21] (difference of 3.12, *T*-value = 9.94, *p*-value = 0.00) for the most significant improvements. This increase in contrast provides evidence that visual improvement may translate into better discrimination between regions and elements in the image, given the increased distance between pixels. On the other hand, the visual comparison presented in Figure 5 shows that the differences, on the contrary, can be perceived as shadow loss between vegetation regions, i.e., [19,21,27] can obtain a better perception of the colors, but lose the shadows between the bright and dark regions of the vegetation. However, the proposed method obtained a slightly reduced mean in the entropy measure (difference = 0.122, *T*-value = 4.37, *p*-value = 0.00). Let us consider that the proposed method fuses the information from NIR, maintaining the relationship between the red, blue, and green channels without focusing on details such as the fusion method of [28]. However, [28] extracts the details with the top-hat transform before the fusion step, which can be included in an extension of the method. In contrast, maintaining the relationship between channels avoided a stronger reduction in entropy than [21], because although the luminance in the image increased, fusion is performed more straightforwardly in this method, which can be seen as a greater difference in the means with the original images (difference = 0.283, *T*-value = 10.06, *p*-value = 0.00). Unlike other fusion strategies, the proposed method does not include information on the ridges, edges, and corrugated areas of the NIR band, i.e., the greenhouse tarpaulin is more corrugated in [21,28]. However, the proposed method remains the same as the original image, because this part was not enhanced and remains the same.

**Figure 7:**
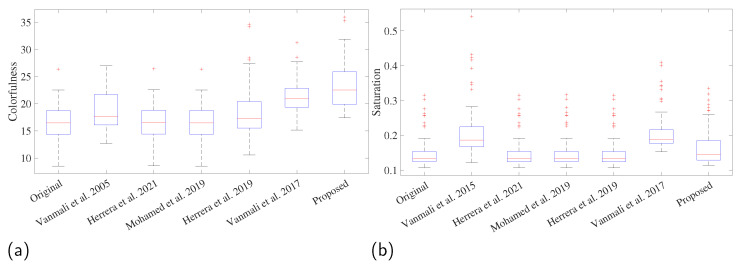
Comparison of image fusion quality metrics among methods. (**a**) Colorfulness, and (**b**) saturation. The methods that were compared are Vanmali et al. 2015 [19], Herrera et al. 2019 [21], Vanmali et al. 2017 [27], Herrera et al. 2021 [28], Mohamed et al. 2019 [29].

However, most of the methods compared significantly improve color, resulting in images with different colors than the original, [19,21,27], and the proposed method consistently changes the color of the images, which can be distinguished perceptually in the comparison presented in Figure 5. The proposed method significantly increased the colorfulness (difference = 6.66, *T*-value = 10.09, *p*-value = 0.00), but compared to the reference method, the saturation remains the same as the original images (difference = 0.0147, *T*-value = 1.46, *p* = 0.766). The change in these two metrics (See Figure 7) can make unripe blackberries look unnatural [19,21,27].

The results show that the proposed method can obtain images with greater contrast between regions. These more colorful images can improve the visual perception of elements in the image without oversaturating the image and without obtaining unnatural colors. These results are because the proposed method controls this aspect with the parameters δ and ω and the transmission map. When the image is more colorful and with more light, the transmission map controls the fusion to take more information from the original image and less from the NIR and vice versa in an adaptive process that can control the added fusion depending on the light in the scene.

However, in addition to image quality, experiments were performed to understand how fusion strategies could be helpful in a classification task between environment and unripe and ripe blackberries. The results between the original image and the fused methods were compared to determine the effect of the strategies on the classification rates. We expect to improve the classification rates in images with low light because the proposed method increases the contrast and colorfulness, which may be helpful to a classification method in circumstances where the light is challenging, such as those in which the images are little exposed or the ambient light is insufficient.

The blackberries in the images were annotated with bounding boxes, a square shape around the fruit, and a label that assigned a maturity stage to the fruit: unripe, nearly ripe, and ripe. The unripe maturity level was assigned to all blackberries with a green or orange color, the nearly ripe stage was assigned to those fruits with a predominant dark color but with orange patches in the image, and the ripe stage considers only those dark fruits without orange spots. Then the square patches of the fruits were cropped and resized to a size of 224 × 224 pixels and classified according to the method used to process them and their maturity stage, obtaining a dataset with 2203 images, depicted in Table 1. The dataset was balanced at the validation to maintain the same proportion in the training and validation sets. Additionally, a data augmentation process was applied to obtain more variability with a random reflection with probability 50%, a random rotation between −180 and 180 degrees, a random translation on the X and Y axes between −15 and 15 px, and a random shear between −10 and 10 px. Data augmentation was applied to the original images prior to each training iteration so that the probability of training with the same image decreases. The images were labeled according to the overall illumination of the scene, and the mean luminance in the CIE L*a*b color space of the images was calculated and classified according it; a threshold of 40 of the mean luminance was used in channel L, dividing the images with low (underexposed) illumination from those with normal illumination. Some images with low and normal illumination before and after processing them with the fusion methods are shown in Figure 5.

Object detection in computer vision is a task that involves locating coordinates for each object of interest in an image, which in our case is blackberries. The goal of a detection model is to locate the boundaries of the object in the image by determining a boundary box around them, which can be defined as {x,y,w,h}, where *x* and *y* are the center of the bounding box and *w* and *h* are the width and height, respectively. The object detection task typically takes an image as input, extracts features that allow the model to identify where an object is in the image, then proposes the possible objects in the image, and the last step classifies the proposals into predefined categories or classes, for example, background, ripe blackberry, or unripe blackberry. Experiments were conducted with the classification stage to extract from the model the influence of the feature extraction capabilities and the region proposal stages of a detection model. In this manner, the analysis performed can help to understand whether the fusion methods work not only in harvester tasks, but also in post-harvesting processes such as classification before sending the fruits to storage or stores. In addition, an experiment was performed to understand if the fusion methods can help to discriminate between background and fruits (the proposal stage) by training and evaluating the classification models with two classes, fruit and no fruit, independently of its ripeness stage. Thus, K-fold validation with K=10 was used for repeatability to validate an SVM model with default parameters for multiclass classification with features extracted from a pre-trained ResNet50 model with ImageNet with frozen weights to avoid introducing variability in the statistical model from the training and feature extraction steps to determine with more confidence the effect of the fusion methods, taking the original image set as a baseline. The databases were divided into fruit and no fruit, in which the unripe, nearly ripe, and ripe blackberries were grouped as positive classes and the images of the background without fruits as negative ones. This experiment allowed us to determine the ability of a classification model to discriminate between blackberries independently of their ripeness and other vegetation, such as leaves, stems, and flowers. This is a difficult task in low-light conditions or when the exposure time of the cameras is not appropriately selected. The results suggest that a classification model trained with images processed with the proposed method performed better for both types of illumination: low and normal.

The classification model achieved F1 score measures of 0.871 and 0.897, respectively, for the two types of illumination (see Table 2). On the contrary, the method with which the classifier model had the lowest performance is Herrera et al. 2019 [21], with 0.518 and 0.491, respectively. This result means an improvement of up to 18 % with respect to the F1-measure obtained with images without treatment as the training dataset for low-illumination conditions (0.736±0.296) and 14% for normal illumination (0.789±0.254). It is important to note that the standard deviation obtained with the data of the proposed method was the lowest of all methods, with 0.085 for low illumination and 0.064 for normal illumination, being the main improvement of the model, obtaining more robust classification rates independently of the light in the scene. On the other hand, the highest standard deviation obtained was 0.372 and 0.356 for low and normal illumination by Vanmali et al. 2017 [27]. Then, to stabilize the effect of the fusion models to distinguish fruits from the unripe and ripe stages, background images without a fruit were discarded and only those with fruits were maintained in this experiment, considering ripe fruits as the positive class and nearly unripe and unripe as the negative class. The classification model achieved the highest F1 score using processed images with the proposed fusion method as input, maintaining the low- and normal-illumination results, at 0.944 and 0.973, respectively. In contrast, the model with the lowest F1 score was Herrera et al. 2019 [21], with 0.841 for low and 0.849 for normal. As in the first experiment, the proposed method proved to be the most robust despite illumination circumstances, allowing the classifier model to obtain the smallest value as the standard deviation, 0.036 at low illumination and 0.019 at normal illumination. Instead, the method with more variation in a low-illumination scenario was Herrera et al. 2021 [28] (0.148), while in normal illumination it was Vanmali et al. 2015 [19] (0.108).

**Figure 8:**
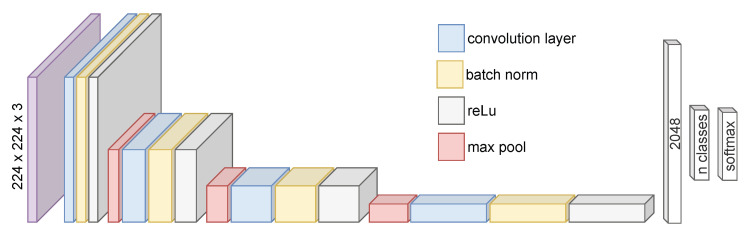
Architecture of the used CNN for the classification of the blackberries.

The results obtained with the proposed method imply a gain of 4.76% and 2.55% improvement in low and normal illumination compared to the model trained with images without processing (0.901±0.112 and 0.948±0.034). Then another experiment evaluated the overall performance of the classification model trained with the images processed for each fusion method. The data used for the training are the complete database considering each separation for the analysis. It consists of four classes: background, unripe blackberry, nearly ripe blackberry, and ripe blackberry. The proposed method obtained a mean F1-score of 0.935 ± 0.060, which corresponds to the highest classification rate with the lowest dispersion, making the proposed method more robust to changes in illumination and improving the classification rates to 7.27% regarding training the classifier method without image fusion. The improvement in mean and dispersion may be due to adaptive enhancement of the image depending on the calculated atmospheric light and the vegetation index that controls the amount of information fused from the enhanced and NIR images. The F-1 score was used to evaluate the classification models because it considers the correct classification of the samples under unbalanced datasets, or when there is only one class of interest and several negative classes, i.e., when evaluating the ability of the classification model to distinguish between unripe, nearly ripe, and ripe fruits and the background independently of their ripeness stage and the background. A Convolutional Neural Network (CNN) architecture was selected to determine the effectiveness of a computational model that performs inference in a limited environment with low computing power and energy constraints. The selected architecture is a common, simple, and effective design for a typical image classification task (see Figure 8). It contains a series of convolutional and batch normalization layers with ReLu activation followed by maximum pooling. This series of layers enables the classifier to learn regularized spatially invariant hierarchical features from the data, which is essential in datasets with light variations, as in our case. The first layer consists of an input layer with a size of 224 × 224, which includes the three channels, red, blue, and green, of the image to be classified. Then the architecture has three consecutive sets of convolutional layers with a filter size of 5 × 5 and max pooling layers with a filter size of 2 × 2. The convolutional layers have an increasing number of filters: eight for the first set, sixteen for the second, and thirty-two for the last convolutional layer. The activation function for the convolutional layers is ReLu, stride 1 for the convolutions, and stride 2 for the max pooling layers. The final convolutional layer has eight filters and a filter size of 5 × 5 to obtain 2048 features connected to a fully connected final layer with five neurons and a Softmax function. The CNN was trained with stochastic gradient descent with momentum optimizer with 100 maximum epochs, a validation patience of 5, a validation frequency every 10 iterations, and a mini-batch size of 32 images per iteration with a hold-out strategy for validation with a set 70% (1542 images) for training and 30% (661 images) for testing.

The results show the effectiveness of the fusion methods in improving classification rates under both light conditions, underexposed and normal illumination, depicted in Table 3. The proposed method consistently obtained the overall highest F1 measurement of the other fusion methods under low- and normal-illumination conditions. It achieved the highest F1 scores for fruit classification and ripeness assessment, achieving 2.23% and 5.72% of improvement in low and normal illumination, respectively, in comparison with the images without processing (0.941 ± 0.081 and 0.926 ± 0.021), suggesting that the method is effective in improving the quality of input images for the frozen ResNet50 and trained CNN model. These results suggest that the fusion methods and more specifically the proposed one can enhance fruit classification rates in challenging conditions, which can impact the reduction in spoiled fruit, improving harvesting efficiency in this application.

Additionally, the images were processed to determine the processing time and frames per second that can be obtained in the MATLAB environment. Although these results could be comparable, let us remark that the time can be improved if the code is implemented in a more efficient language code and the obtained values are used for reference in the comparison. The average execution time of the proposed method was 0.064±0.003 s, which means at least 15 fps compared to 2 fps for Herrera et al. 2019 ([21]) and Herrera et al. 2021 ([28]), 1 fps for Vanmali et al. 2017 ([27]), 1 fps for Vanmali et al. 2015 ([19]), and 0.06 fps for Mohamed et al. 2019 ([29]). This number of frames per second means that the method is 7 times faster than Herrera et al. 2019 and Herrera et al. 2021 [21,28], 13 times faster than Vanmali et al. 2017 [27], and 249 times faster than Mohamed et al. 2019 [29]. The mean time for all methods is presented in Table 4 along with a *T*-value and a *p*-value of multiple comparisons of Tukey with α=0.05.

## 4. Conclusions and Future Work

Many sensors can add valuable information to a visible image, i.e., the NIR spectral band preserves information less affected by haze fog or poor light conditions than the visible spectrum. It may also provide information about the metabolism of vegetation when processes are carried out along the red band of an RGB image. The image fusion techniques aim to combine information between visible spectral bands and those that can add information to an image that can be interpretable by a human being; for these reasons, the information fusion between VIS and NIR images of blackberries in a greenhouse was explored in order to improve the classification rates between ripe and unripe fruits.

The experiments show that the proposed method can process images in 64 ± 3 ms; this means that the method can process up to 15 frames per second, making its use viable before a classification or detection task when applicable. On the other hand, the analysis of the image fusion metrics shows that the proposed method may improve the contrast and colorfulness of low-exposure images while the saturation is kept the same as the original, but the reduction in the entropy in the image is the main drawback. This improvement in image contrast makes fruits more perceptible in low-light conditions, improving blackberry classification rates in the wild under challenging uncontrolled light. The improvement in classification rates and the reduction in standard deviation mean a step closer to the development of a robust automated harvesting robot under different illuminations, achieving 18% and 14% of F1-score value improvement in low and normal illumination, respectively, for binary fruit vs. no fruit classification, as well as 4.76% and 2.55% with a pre-trained model for multi-class classification, and 2.23% and 5.72% with a trained model, all of them in comparison with the images without processing as the training set. The next step of this work may be to analyze the effect of image fusion in blackberry detection under low-light conditions and the use of the vegetation index to detect objects or obstacles for the robotic arm that can be more easily identified in the NIR band, such as the guidelines used to keep plants upright.

## Figures and Tables

**Table 1:** Database description.

No Fruit	Unripe	Nearly Ripe	Ripe	Total
419	1480	98	206	2203

**Table 2:** Comparison of F1-score obtained with a pretrained ResNet50 model.

Low Illumination			
**Fusion Method**	**Fruit vs. No Fruit**	**Ripe vs. Unripe**	**Total**
[19]	0.649 ± 0.329	0.903 ± 0.089	0.790 ± 0.262
[27]	0.663 ± 0.372	0.892 ± 0.107	0.777 ± 0.297
[29]	0.619 ± 0.308	0.858 ± 0.118	0.752 ± 0.253
[21]	0.518 ± 0.329	0.841 ± 0.132	0.697 ± 0.289
[28]	0.716 ± 0.328	0.905 ± 0.148	0.821 ± 0.262
Original	0.736 ± 0.296	0.901 ± 0.112	0.818 ± 0.238
Proposed	0.871 ± 0.085	0.944 ± 0.036	0.909 ± 0.074
**Normal Illumination**			
[19]	0.887 ± 0.083	0.887 ± 0.108	0.887 ± 0.098
[27]	0.747 ± 0.356	0.889 ± 0.103	0.821 ± 0.266
[29]	0.892 ± 0.110	0.908 ± 0.097	0.901 ± 0.103
[21]	0.491 ± 0.317	0.849 ± 0.105	0.690 ± 0.287
[28]	0.819 ± 0.211	0.949 ± 0.087	0.896 ± 0.164
Original	0.789 ± 0.254	0.948 ± 0.034	0.873 ± 0.194
Proposed	0.897 ± 0.064	0.973 ± 0.019	0.935 ± 0.060

**Table 3:** Comparison of F1-score obtained with a trained CNN model.

Low Illumination			
**Fusion Method**	**Fruit vs. No Fruit**	**Ripe vs. Unripe**	**Total**
[19]	0.857	1.000	0.929 ± 0.071
[27]	0.932	0.991	0.961 ± 0.029
[29]	0.886	0.991	0.924 ± 0.057
[21]	0.825	0.980	0.903 ± 0.078
[28]	0.854	0.980	0.917 ± 0.063
Original	0.829	0.974	0.941 ± 0.081
Proposed	0.933	0.991	0.962 ± 0.028
**Normal Illumination**			
[19]	0.914	0.946	0.930 ± 0.016
[27]	0.921	0.962	0.941 ± 0.021
[29]	0.921	0.962	0.951 ± 0.030
[21]	0.846	0.985	0.915 ± 0.069
[28]	0.900	0.991	0.946 ± 0.046
Original	0.897	0.995	0.926 ± 0.021
Proposed	0.958	1.000	0.979 ± 0.029

**Table 4:** Comparison of processing time.

Fusion Method	Processing Time	T-Value	*p*-Value
[19]	1.076±0.043	−187.47	0.00
[27]	0.864±0.013	−269.80	0.00
[29]	15.940±0.130	−4317.04	0.00
[21]	0.459±0.005	−90.00	0.00
[28]	0.489±0.008	−97.24	0.00
Proposed	0.064 ± 0.003	N/A ^1^	N/A ^1^

N/A ^1^: Not applicable.

## Data Availability

The data presented in this study are available on request from the corresponding author.
